# Epidemiological and Clinical Characteristics of Bronchiolitis and the Impact of RSV Infection: A Five-Year Study in a Tertiary Pediatric Center in Central Romania

**DOI:** 10.3390/pediatric18030075

**Published:** 2026-06-02

**Authors:** Alexandra-Antonela Obaciu, Veronica Purdel, Laura Bleotu, Vlad Monescu, Mariana-Alexandra Grecu, Ioana Arbanas, Oana Falup-Pecurariu

**Affiliations:** 1 Faculty of Medicine, Transilvania University of Brașov, 500019 Brașov, Romania; alexandra.salvar@gmail.com (A.-A.O.); lazarmalexandra@gmail.com (M.-A.G.); ioana.arbanas@yahoo.com (I.A.); 2 Children’s Emergency Clinic Hospital, 500063 Brașov, Romania; laurableotu@yahoo.com; 3 Pfizer Romania SRL, Vaccines, Willbrook Platinum Business & Convention Center Sos, 013686 Bucharest, Romania; veronica.purdel@pfizer.com; 4 Faculty of Mathematics and Computer Science, Transilvania University of Brașov, 500091 Brașov, Romania; monescu@gmail.com

**Keywords:** bronchiolitis, respiratory syncytial virus, RSV, infants, hospitalization, seasonality, disease severity

## Abstract

Background: Bronchiolitis, due to respiratory syncytial virus, is the most common cause of hospitalization and lower respiratory tract infections in infants and toddlers across the globe. Data on RSV epidemiology in Romania are limited and are mainly derived from national surveillance systems. Understanding regional trends in RSV bronchiolitis, its etiology, and its severity is important while assessing the potential impacts of future prevention measures. Methods: We conducted a retrospective cohort study including infants hospitalized with bronchiolitis between 2019 and 2023 in a tertiary pediatric center in Central Romania. Demographic, clinical, and treatment data were analyzed. Comparative analyses between RSV-positive and RSV-negative cases were performed among patients tested for RSV. Disease severity was assessed using oxygen saturation at admission and length of hospital stay. Results: A total of 2967 bronchiolitis hospitalizations were identified during the study period. After exclusion of 167 cases due to hospitalization <24 h or incomplete medical records, 2800 patients were included in the final analysis. The number of admissions decreased in 2020 (*n* = 301) compared to 2019 (*n* = 638), followed by an increase in 2021 (*n* = 463) and a peak in 2022 (*n* = 745), with a slight decrease in 2023 (*n* = 653). Among tested patients, RSV positivity increased from 14.4% in 2019 to 37.7% in 2022, then decreased to 27.4% in 2023. RSV-positive cases were more frequent in younger age groups, particularly those under 6 months of age. Compared to RSV-negative cases, RSV-positive bronchiolitis was associated with lower oxygen saturation at admission, and a longer hospital stay (<0.001), indicating a more severe clinical course. Treatment differences were also observed, with higher use of corticosteroids in RSV-positive patients (*p* < 0.002), while antibiotic use was similar between groups (*p* = 0.149). Conclusions: RSV infection was associated with a more severe clinical course in our cohort and continues to play a central role in the burden of disease. The variability observed in treatment practices also suggests that further efforts are needed to better align clinical management with current evidence-based recommendations.

## 1. Introduction

Bronchiolitis, due to respiratory syncytial virus, is the most common cause of hospitalization and lower respiratory tract infection in infants and toddlers across the globe [1]. RSV is an enveloped, negative-sense, single-stranded RNA virus of the Orthopneumovirus genus that typically causes seasonal winter epidemics in temperate climates [2]. Understanding regional trends of RSV bronchiolitis including its etiology and severity is important to assess the impact of future prevention measures [3], as most epidemiological studies are focusing on hospitalization [4].

In Romania, data on RSV epidemiology remains limited and are mainly derived from national surveillance systems. Available reports from the 2022–2023 season include RSV detection primarily in the context of co-infections with other respiratory viruses, such as influenza (77 cases) and SARS-CoV-2 (76 cases), rather than as a distinct, systematically reported pathogen. As a result, these data do not provide a clear estimate of the burden of RSV-associated bronchiolitis in the pediatric population [5].

Clinical manifestations of RSV infection range from mild upper respiratory tract symptoms to severe lower respiratory tract disease, including bronchiolitis and pneumonia. Although most children are infected by two years of age, severe diseases occur disproportionately in young infants, particularly those under six months of age [6]. Bronchiolitis requiring hospitalization is caused by several viral agents; however, RSV accounts for up to 80% of hospitalized bronchiolitis cases [7]. Diagnosis is primarily clinical, with laboratory confirmation—using rapid antigen testing or nucleic acid amplification techniques (PCR)—reserved for select cases [8].

Treatment of RSV infection is mainly supportive, including oxygen supplementation and hydration.

The relationship between RSV bronchiolitis and later respiratory morbidity, including recurrent wheezing and asthma, remains a subject of ongoing debate [9,10].

Several prophylactic measures are in use and are recommended by the American Academy of Pediatrics.

One of the recommended prophylactic measures includes palivizumab, a monoclonal antibody against respiratory syncytial virus (RSV) for RSV prophylaxis in infants and young children who are at increased risk of hospitalization, such as those born before 29 weeks’ gestation, children with chronic lung disease from prematurity, or significant congenital heart disease. Palivizumab is administered as monthly intramuscular injections during the RSV season. This recommendation is based on evidence of reduced RSV hospitalization rates in these high-risk groups [11].

Although palivizumab has been available in Europe since 1999, its structured use within the Romanian healthcare system, based on a national protocol, was only implemented in 2022; therefore, during the study period (2019–2023), no patients in our cohort received RSV prophylaxis with palivizumab [12].

In addition to palivizumab, other preventive strategies against RSV infection are available, including long-acting monoclonal antibody-based and maternal vaccination during pregnancy.

Nirsevimab, a monoclonal antibody targeting the prefusion RSV F protein, has been authorized in Europe in recent years for the prevention of respiratory syncytial virus infection in infants; in large clinical trials, it has been shown to significantly reduce RSV related lower respiratory tract infections [13]. In Romania, nirsevimab has undergone health technology assessment, reflecting the initial steps toward inclusion in national prevention strategies.

However, nirsevimab prophylaxis and maternal RSV vaccination are not yet part of Romania’s national prevention programs, and their elective administration is not routinely reimbursed. Their administration depends on physician recommendation and individual access, as they are generally obtained outside publicly funded healthcare schemes.

During the study period (2019–2023), this strategy was not part of routine clinical practice in Romania, and its implementation remains recent [14].

Maternal vaccination has also emerged as a preventive strategy, with recent studies showing high efficacy in reducing severe RSV-associated lower respiratory tract infections in early infancy [15].

The Society for Maternal–Fetal Medicine recommends that all infants be protected against RSV-associated lower respiratory tract infection through either maternal vaccination during pregnancy or direct infant immunization with a monoclonal antibody [16].

These preventive strategies reflect the significant burden of RSV infection in early childhood, which is also supported by the clinical severity and hospitalization patterns observed in our cohort.

Given the limited epidemiological data available for Romania and the absence of systematic RSV testing in most pediatric units, the true burden of RSV-associated bronchiolitis remains largely underestimated. This gap in knowledge highlights the need for institution-based studies capable of providing detailed clinical data, including disease severity, hospitalization outcomes, and etiological confirmation.

The aim of this study is to provide a comprehensive overview of the epidemiological characteristics, age-related patterns, and hospitalization outcomes of infants and young children admitted for bronchiolitis in a tertiary hospital in the center of Romania.

A secondary objective was to compare clinical characteristics and hospitalization outcomes between RSV-positive and RSV-negative bronchiolitis among tested admissions.

## 2. Materials and Methods

### 2.1. Study Design and Population

We conducted a single-center retrospective cohort study at the Clinical Emergency Hospital for Children, Brasov, Romania, covering a five-year period from January 2019 to December 2023. The study included all bronchiolitis-related hospital admissions aged 0–24 months during the study period, regardless of potential readmissions.

### 2.2. Patient Selection

Patients were identified based on hospital discharge diagnoses of bronchiolitis. All hospital admissions with a clinical diagnosis of bronchiolitis were considered eligible for inclusion.

The unit of analysis was the hospitalization episode rather than the individual patient; therefore, repeat admissions for the same patient may have been included. No sensitivity analysis, excluding readmissions, was performed.

Exclusion criteria included hospitalization duration <24 h and incomplete or missing medical records.

### 2.3. RSV Testing and Classification

RSV status was defined based on available laboratory testing. RSV testing was performed selectively, according to clinical indication and test availability.

For analyses involving RSV status, only patients with available RSV test results were included and classified as RSV-positive or RSV-negative.

Patients without RSV testing were included in the overall cohort description but were not considered in analyses comparing RSV-positive and RSV-negative groups, as their RSV status could not be determined.

For rapid detection of the RSV antigen, the Standard Q RSV Ag Test (SD Biosensor Suwon, Republic of Korea), an immunochromatographic method using nasopharyngeal swab samples, was utilized. These antigenic tests were especially used at the admission of patients in the emergency department.

Among the tests performed for the detection of RSV infection was Xpert Xpress CoV-2/Flu/RSV Plus (REF XP3COV2/FLU/RSV-10, Cepheid, Sunnyvale, CA, USA), areal time PCR method.

Rapid antigen testing was the primary diagnostic method used in routine clinical practice, while multiplex PCR was performed in a limited number of cases.

### 2.4. Disease Severity Assessment

Disease severity was assessed based on routinely documented clinical parameters, including oxygen saturation at admission, the need for supplemental oxygen, and the length of hospital stay.

### 2.5. Data Collection

We collected variables such as demographic characteristics (age, sex, environment, gestational age), clinical variables (oxygen saturation at admission, duration of symptoms prior admission, length of stay) and treatment-related variables, including use of systemic corticosteroids, antibiotics, oxygen supplementation, and the type of inhaled therapy used (saline, salbutamol, epinephrine).

The classification of rural versus urban residence was based on the patients’ home address, using the official administrative classification of localities in Romania.

### 2.6. Informed Consent Statement

The study protocol was reviewed and approved by the Ethics Committee of the Children’s Clinical Emergency Hospital, Brașov (Approval Code: 9/4591; Approval Date: 13 March 2025), in accordance with the Declaration of Helsinki. Written informed consent was obtained from all parents or legal guardians prior to inclusion of anonymized medical data in the study.

### 2.7. Statistical Analysis

The statistical analysis was performed using Python (version 3.11.6, Python Software Foundation, Wilmington, DE, USA) in a Jupyter Notebook environment (version 7.2.2., Project Jupyter, Berkeley, CA, USA). Data processing and statistical analyses were conducted using the following libraries: pandas (version 2.2.2, NumFOCUS, Austin, TX, USA), NumPy (version 1.26.4, NumFOCUS, Austin, TX, USA), and SciPy (version 1.17.1, NumFOCUS, Austin, TX, USA). Continuous variables were summarized as mean and standard deviation when data were normally distributed, or as median and interquartile range (IQR) when distributions were skewed. Categorical variables are presented as numbers and percentages.

The primary outcome analyzed was the length of hospital stay (LOS), measured in days.

As LOS was not normally distributed, nonparametric tests were used. Comparisons between groups (such as RSV-positive versus RSV-negative bronchiolitis,) were performed using the Mann–Whitney U test. Comparisons between more than two groups (for example, age groups) were made using Kruskal–Wallis test.

Differences between categorical variables were assessed using the Chi-square test or Fisher’s exact test, depending on the data. Treatment use (oxygen therapy, nebulized therapy, corticosteroids, and antibiotics) was analyzed across age groups, gestational age categories, and in relation to associated conditions.

Length of hospital stay is reported as median and interquartile range (25th–75th percentiles). All tests were two-sided, and a *p*-value < 0.05 was considered statistically significant.

## 3. Results

A total of 2967 infants and young children were hospitalized with bronchiolitis during the study period. After applying the inclusion and exclusion criteria, 2800 patients were included in the final analysis, while 167 cases were excluded due to short hospitalization (<24 h) or incomplete medical records. Because of the retrospective design, we could not reliably determine the exact number of exclusions for each individual criterion.

Of the total bronchiolitis cohort, RSV testing was available in 1572 patients. Analyses comparing RSV-positive and RSV-negative cases were limited to this tested subgroup, while untested patients were included only in descriptive analyses of the overall cohort.

### 3.1. Baseline Characteristics of the Overall Cohort

**Figure 1:**
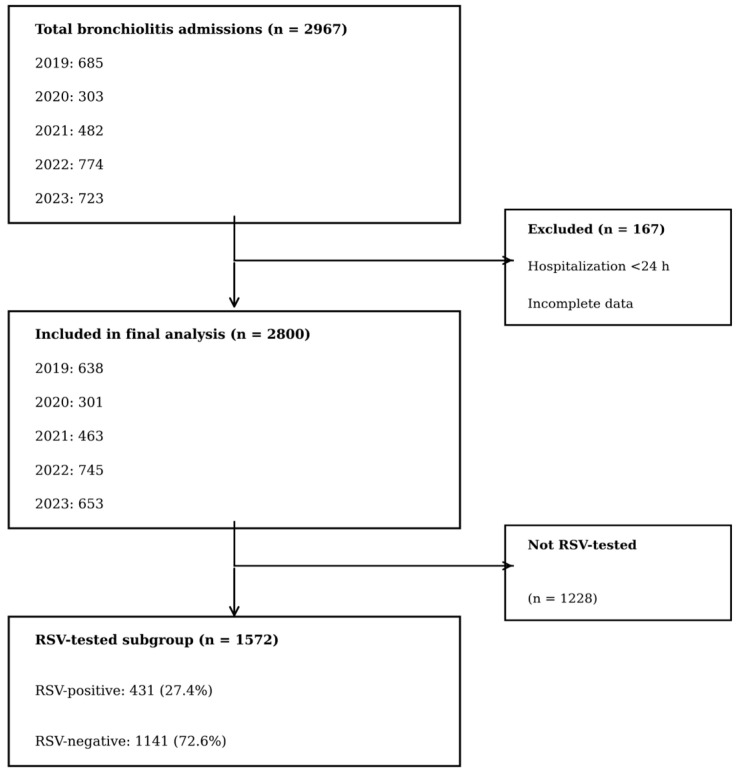
Flow diagram of patient selection.

The patient-selection process is summarized in Figure 1. The baseline characteristics of the study population are presented in Table 1.

Figure 1 illustrates the total number of bronchiolitis admissions identified during the study period, the number of patients included in the final analysis, and their yearly distribution, as well as the RSV-tested subgroup.

The median age at admission was 4 months (IQR 2–9), with a mean age of 6.2 ± 5.9 months. Male patients accounted for 60.3% of the cohort. Preterm patients were documented in 17.9% of cases. Most patients originated from rural areas (61.9%), while 38.1% were from urban environments.

### 3.2. Seasonal Trends

**Figure 2:**
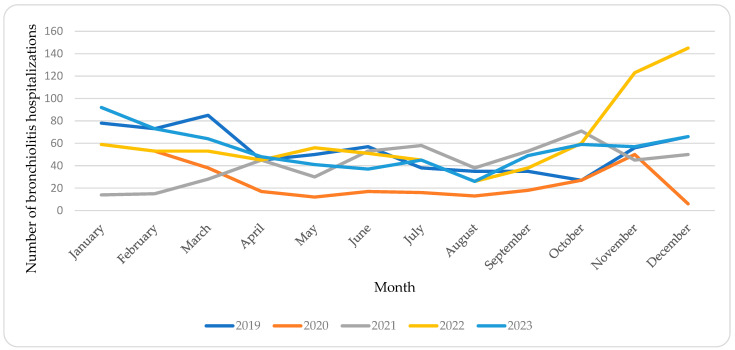
Annual and seasonal trends in bronchiolitis hospitalizations (2019–2023).

Seasonal variation of bronchiolitis hospitalizations is illustrated in Figure 2. Admissions showed a clear seasonal pattern, with peaks observed during the winter months, particularly in November and December, while the lowest number of hospitalizations occurred during the summer months. A marked reduction in the number of hospitalizations was observed during the COVID-19 pandemic period in 2020, followed by a progressive increase in subsequent years. In addition, compared to previous years, a higher number of bronchiolitis hospitalizations was observed during the summer months in 2022 and 2023.

This figure illustrates the monthly distribution of bronchiolitis hospitalizations across the study period. The x-axis represents calendar months, and the y-axis shows the number of hospitalizations. A clear seasonal pattern is observed, with peaks during the winter months. Differences between years are also visible, including a marked reduction in 2020 and higher case numbers in 2022 and 2023, including during summer months.

### 3.3. Age Group Comparisons and Hospitalization Outcomes

**Figure 3:**
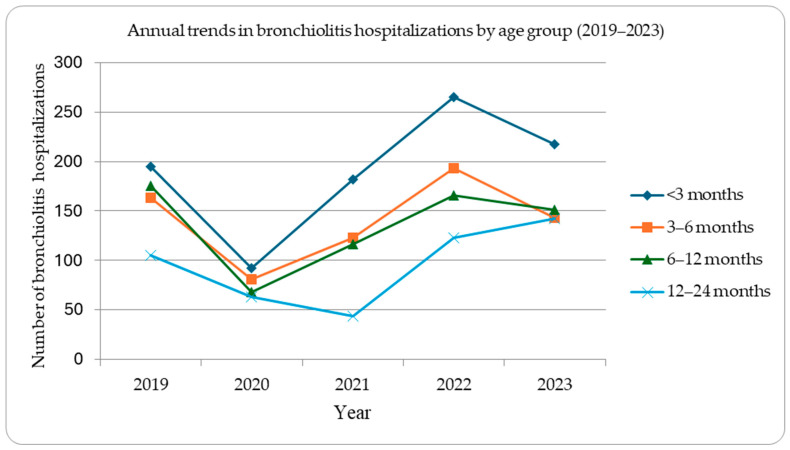
Annual trends in bronchiolitis hospitalizations by age group (2019–2023).

The distribution of bronchiolitis hospitalizations across age groups during the study period is illustrated in Figure 3.

The clinical characteristics of bronchiolitis according to age group are presented in Table 2.

This figure shows the yearly distribution of bronchiolitis hospitalizations stratified by age group (<3 months, 3–6 months, 6–12 months, and 12–24 months). The x-axis represents the study years, and the y-axis indicates the number of hospitalizations. Infants younger than 3 months consistently accounted for the highest number of admissions across all years.

When we analyzed the cohort characteristics by age group, we saw that the need for oxygen therapy increased progressively with age (*p* < 0.001). Wheezing was more common in older infants and young children, whereas younger infants had longer hospital stays (Table 2).

Table 3 summarizes hospitalization outcomes and treatment. Oxygen saturation below 92% at admission was observed in 10.4% of patients. The median hospital stay was 7 days (IQR 5–9), and 32.0% required oxygen therapy. Nebulized therapy was widely used (90.5%), along with systemic corticosteroids (85.1%) and antibiotics (49.3%).

### 3.4. Comparison Between RSV-Positive and RSV-Negative Bronchiolitis

Among the study population, 1572 patients underwent RSV testing and were included in the subgroup analysis. The baseline characteristics according to RSV status are presented in Table 4. Age and prematurity were similar between groups, while male sex and rural residence were more common in RSV-negative patients.

**Figure 4:**
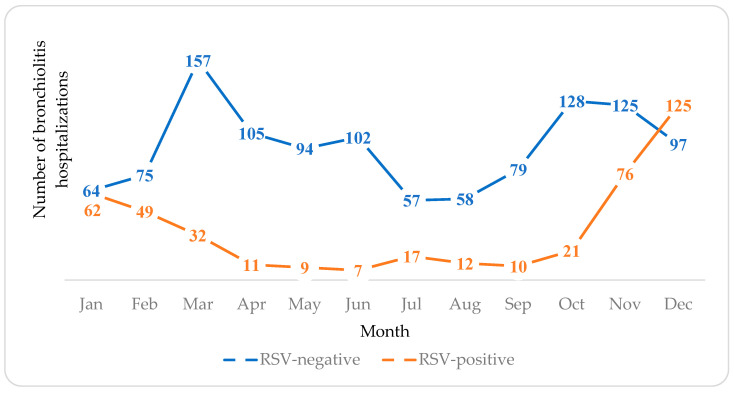
Monthly distribution of bronchiolitis hospitalizations by RSV status.

Monthly distribution of bronchiolitis cases among patients tested for RSV infection are represented in Figure 4. RSV-positive cases showed a clear peak during the winter months, particularly in November and December, whereas RSV-negative cases were more evenly distributed throughout the year.

Differences in clinical courses were observed between RSV-positive and RSV-negative bronchiolitis (Table 5). RSV-positive cases were associated with a higher frequency of hypoxemia at admission, a greater need for oxygen therapy, and a slightly longer duration of hospitalization. The use of systemic corticosteroids was more frequent in RSV-positive patients, whereas antibiotic use was comparable between the two groups.

Additional analyses of length of hospital stay according to age group and treatment are presented in the Supplementary Materials (Tables S1 and S2; Figures S1 and S2).

## 4. Discussion

This single-center retrospective cohort study provides a comprehensive overview of bronchiolitis hospitalizations in a tertiary pediatric center over a five-year period, with a particular focus on the clinical and epidemiological differences associated with RSV infection.

In our cohort, RSV-bronchiolitis had a more severe clinical presentation compared to RSV-negative bronchiolitis, as shown by lower oxygen saturation at admission and a longer hospital stay. These findings are consistent with previous studies demonstrating that RSV is one of the main drivers of severe lower respiratory tract infections in infants and young children [17,18].

However, this finding should be interpreted with caution, as RSV testing was performed selectively, and more severe cases may have been more likely to be tested. This may have contributed to an overestimation of the association between RSV positivity and disease severity.

Notably, most hospitalizations occurred in full-term infants without underlying medical conditions, despite the well-established role of prematurity and chronic comorbidities as risk factors for severe disease. Our observations are in line with previously published data [19,20,21], establishing RSV as the principal driver of severe lower respiratory tract disease in infants, likely due to its tropism for the lower airways and the increased inflammatory response in early life.

In our study, most RSV-positive hospitalizations occurred in infants younger than six months, with a clear predominance in the first three months of life. This pattern of age distribution aligns with findings from large multicenter studies conducted across Europe [22,23,24,25], where more than half of RSV hospitalizations involved infants below three months of age, regardless of season or country. We also found younger infants had longer hospital stays, especially those under one month. Although the length of stay reported in the BRICE study and other European cohorts [21,26,27], the same age-related pattern was observed. This difference may be explained by local admission criteria, discharge policies, healthcare system organization.

From an epidemiological perspective, we also observed a clear seasonal pattern, with a peak in bronchiolitis hospitalizations during the winter months. This is in line with the well-described seasonal circulation of respiratory viruses, particularly RSV, in temperate climates. A substantial reduction in admissions was observed in 2020, followed by a progressive increase in subsequent years and a peak in 2022. In parallel, the proportion of RSV-positive cases among tested admissions increased over time, reaching its highest level in 2022 before declining in 2023.

Consistent with our findings, post-COVID-19 studies described a marked decline in RSV cases during the pandemic, followed by increased RSV-related hospital admissions in 2021 and changes in seasonal trends [28,29,30]. These changes that occurred after the lifting of restrictions were explained by studies as a concept of immunity debt, meaning that reduced exposure to respiratory viruses during the pandemic may have led to a larger susceptible population after restrictions were lifted [31,32,33]. In clinical practice, this may translate into less predictable seasonal peaks and a potential increase in the number of susceptible infants presenting with more severe disease after the relaxation of public health measures. In addition, we observed a higher number of cases during the summer months in 2022 and 2023, suggesting a shift from the typical seasonal pattern. This finding may reflect altered viral circulation in the post-pandemic period.

In contrast, RSV-negative bronchiolitis did not follow the same clear winter pattern seen in RSV-positive cases. Instead, case numbers were more spread out across the year, with notable peaks in early spring and autumn. This pattern may be explained by the involvement of different respiratory viruses, each with its own circulation pattern.

In our cohort, the most prescribed medications were systemic steroids (85.1%) and antibiotics (49.3%). Even though bronchiolitis has a viral cause and current guidelines recommend mainly supportive management in bronchiolitis, their use remains common in clinical practice in Romania.

Several studies have reported antibiotic prescription rates ranging from 41.3% in Belgium (in a study conducted by Proesmans M et al. [34]) to 49% in the Netherlands [35]; the highest rates of antibiotic use in bronchiolitis have been reported in Italy, exceeding 70% [36].

A similar pattern is observed for systemic corticosteroids. The rates observed in our cohort, particularly for corticosteroid use, are higher than those commonly reported in the literature.

Previous studies, such as that by Mazela J et al., conducted in Poland [37], who focused on treatment patterns in RSV-infected children, reported 44.3% systemic steroid therapy. Also, a study conducted by Ferronato AE et al. [38] shows high frequencies of the corticosteroids use (52%) for the initial treatment of infants with bronchiolitis; in a retrospective multicenter study, Delaney et al. found an overall rate of 37.5%, with values exceeding 60% in some centers [39].

These findings may be explained by variation in local practice, individual clinical judgment, perceived illness severity, and the presence of wheezing or asthma-like symptoms, which may influence treatment decisions despite guideline recommendations.

When comparing RSV-positive and RSV-negative cases, some differences in treatment patterns became apparent. Systemic corticosteroids were used more often in RSV-positive patients, which may reflect the tendency to treat more severe clinical presentations. In contrast, antibiotic use was similar between the two groups, suggesting that the decision to initiate antibiotics may be driven more by clinical judgment, such as suspicion of bacterial co-infection or diagnostic uncertainty, rather than by RSV status alone. Overall, treatment patterns likely reflect disease severity rather than therapeutic efficacy.

These findings highlight the gap between guideline recommendations and real-world clinical practice. This discrepancy may reflect real-world clinical challenges, including uncertainty in differentiating viral from bacterial infection and the presence of wheezing or asthma-like features that influence treatment decisions.

The gap between guideline recommendations and routine clinical practice points to the need for improved adherence to evidence-based management and stronger antimicrobial stewardship. Measures such as clinician education and the use of clear local protocols could help reduce unnecessary treatments.

The lack of widespread RSV preventive strategies during the study period may partly explain the burden of hospitalization observed in our cohort. As these preventive measures become more accessible, future studies may show changes in disease patterns and severity.

Overall, our findings emphasize the importance of strengthening RSV surveillance and prevention strategies, as well as improving epidemiological monitoring to better guide clinical practice.

### Strengths and Limitations

Several limitations should be considered when interpreting our findings. First, we did not use a validated bronchiolitis severity score, and severity was instead approximated based on routinely documented clinical parameters. Although this reflects real-life clinical practice, it may have reduced the accuracy of severity assessment and limits comparability with studies that rely on standardized scoring systems.

RSV testing in our study was performed selectively, based on clinical indication and test availability. As a result, the tested subgroup may not be fully representative of the overall bronchiolitis population, with a possible overrepresentation of more severe or clinically suspicious cases. This may have led to an overestimation of the association between RSV positivity and disease severity.

Regarding RSV diagnosis, most patients were tested using rapid antigen assays, while PCR confirmation was available only in a small number of cases. Given the lower sensitivity of antigen-based methods, some RSV infections were likely not identified, which may have contributed to the lower proportion of RSV-positive cases observed. Viral testing was not performed in all patients, and RSV status was missing for a considerable part of the cohort.

Finally, the retrospective design of our study comes with several limitations. The unit of analysis was the hospitalization episode rather than the individual patient, so some children may have been included more than once in case of repeated admissions. Missing or incomplete data are also inherent to retrospective studies.

Despite these limitations, our study has several strengths. It includes a relatively large cohort of hospitalized children collected over a five-year period, allowing the evaluation of temporal trends, including changes observed during and after the COVID-19 pandemic. The availability of routinely collected clinical data enabled the assessment of disease severity and treatment patterns in a real-world setting. In addition, the comparison between RSV-positive and RSV-negative cases offers a clearer perspective on the clinical impact of RSV infection in routine practice within a tertiary care setting.

## 5. Conclusions

In conclusion, bronchiolitis remains a common cause of hospitalization, particularly among young infants, and shows a clear seasonal pattern. RSV infection was associated with a more severe clinical course in our cohort, while treatment patterns reflected variability in real-world practice. These findings highlight the importance of careful interpretation of RSV status in the context of selective testing and support the need for improved adherence to evidence-based management strategies. They also underline the importance of continued efforts to improve RSV surveillance and prevention strategies.

## Figures and Tables

**Table 1:** Baseline characteristics of the overall bronchiolitis cohort.

Characteristic	Overall Cohort (*n* = 2800)
Age at admission, months—median (IQR)	4 (2–9)
Age at admission, months—mean ± SD	6.2 ± 5.9
Male, *n* (%)	1689 (60.3)
Female, *n* (%)	1111 (39.7)
Preterm patients (<37 weeks), *n* (%)	500 (17.9)
Term patients (≥37 weeks), *n* (%)	2234 (79.8)
Urban residence, *n* (%)	1067 (38.1)
Rural residence, *n* (%)	1733 (61.9)

**Table 2:** Clinical characteristics of bronchiolitis according to age group.

Characteristic	<3 Months (*n* = 1212)	3–6 Months (*n* = 619)	6–12 Months (*n* = 553)	12–24 Months (*n* = 416)	*p* Value
Length of hospital stay, days—median (IQR)	7 (5–10)	7 (5–9)	6 (5–8)	6 (4–7)	<0.001
Oxygen therapy required, *n* (%)	307 (25.3)	214 (34.6)	202 (36.5)	173 (41.6)	<0.001
Wheezing, *n* (%)	267 (22.0)	212 (34.2)	197 (35.6)	159 (38.2)	<0.001
Crackles, *n* (%)	135 (11.1)	103 (16.6)	109 (19.7)	87 (20.9)	<0.001

**Table 3:** Clinical severity, management, and hospitalization outcomes of the overall cohort.

Variable	Value
Severity/outcomes	
Oxygen saturation at admission < 92%, *n*/N (%)	276/2662 (10.4)
Oxygen saturation at admission ≥ 92%, *n*/N (%)	2386/2662 (89.6)
Length of hospital stay, days—median (IQR)	7 (5–9)
Oxygen therapy during hospitalization, *n* (%)	897 (32.0)
Therapies administered during hospitalization	
Nebulized therapy, any, *n* (%)	2533 (90.5)
Nebulized normal saline, *n* (%)	1143 (40.8)
Nebulized bronchodilator, *n* (%)	1134 (40.5)
Nebulized epinephrine, *n* (%)	411 (14.7)
Systemic corticosteroid therapy, *n* (%)	2384 (85.1)
Antibiotic therapy, *n* (%)	1381 (49.3)

**Table 4:** Baseline characteristics according to RSV status.

Variable	RSV-Negative (*n*= 1141)	RSV-Positive (*n*= 431)	*p* Value
Age (months), median (IQR)	4 (2–8)	4 (2–7)	0.178
Male sex, *n* (%)	723 (63.4)	248 (57.5)	0.039
Female sex, *n* (%)	418 (36.6)	183 (42.5)	
Preterm birth (<37 weeks), *n* (%)	199 (17.4)	78 (18.1)	0.818
Rural residence, *n* (%)	723 (63.4)	244 (56.6)	0.017
Urban residence, *n* (%)	418 (36.6)	187 (43.4)	

**Table 5:** Clinical severity and hospitalization outcomes according to RSV status.

Variable	RSV-Negative (*n* = 1141)	RSV-Positive (*n* = 431)	*p* Value
SpO_2_ <92%, *n* (%)	102 (8.9)	77 (17.9)	<0.001
Oxygen therapy, *n* (%)	346 (30.3)	181 (42.0)	<0.001
Length of stay, days—median (IQR)	7 (5–9)	8 (6–10)	<0.001
Systemic corticosteroid therapy, *n* (%)	1027 (90.0)	412 (95.6)	0.002
Antibiotic therapy, *n* (%)	513 (45.0)	212 (49.2)	0.149

## Data Availability

The data presented in this study are available on request from the corresponding author. The data are not publicly available due to privacy restrictions.

## Supplementary Materials

The following supporting information can be downloaded at: https://www.mdpi.com/article/10.3390/pediatric18030075/s1, Table S1: Length of hospital stay by age group and RSV status (RSV-tested patients only); Table S2: Length of hospital stay according to treatment administered; Figure S1: Length of hospital stay by age group and RSV status among RSV-tested patients; Figure S2: Length of hospital stay according to treatment administered.

## Abbreviations

The following abbreviations are used in this manuscript:

RSV	Respiratory syncytial virus
IQR	Interquartile range
LOS	Length of hospital stay
PCR	Polymerase chain reaction
SpO_2_	Peripheral oxygen saturation
SD	Standard deviation
COVID-19	Coronavirus disease 2019
RNA	Ribonucleic acid
SARS-CoV-2	Severe acute respiratory syndrome coronavirus 2
