# Supplementary material for: Epidemiological and Clinical Characteristics of Bronchiolitis and the Impact of RSV Infection: A Five-Year Study in a Tertiary Pediatric Center in Central Romania
Source: Pediatr Rep. 2026 Jun 2;18(3):75. doi: 10.3390/pediatric18030075 (PMC13305041; doi:10.3390/pediatric18030075)
Supplement: Supplementary file 1 [file pediatrrep-18-00075-s001.zip › Supplementary_Material_MDPI.pdf]

Supplementary Material

Epidemiological and Clinical Characteristics of Bronchiolitis and the Impact of RSV Infection: A Five-Year Study in a Tertiary Pediatric Center

Table S1. Length of hospital stay by age group and RSV status (RSV-tested patients only).

Age Group (months)	RSV-negative, LOS median (IQR)	RSV-positive, LOS median (IQR)
<3 months	7 (5–9)	8 (6–10)
3–6 months	7 (5–9)	8 (6–10)
6–12 months	6 (5–8)	7 (5–9)
12–24 months	6 (4–7)	7 (5–9)

Note: *LOS: length of hospital stay, expressed as median (interquartile range). Analysis includes only RSV-tested patients (n = 1572).*

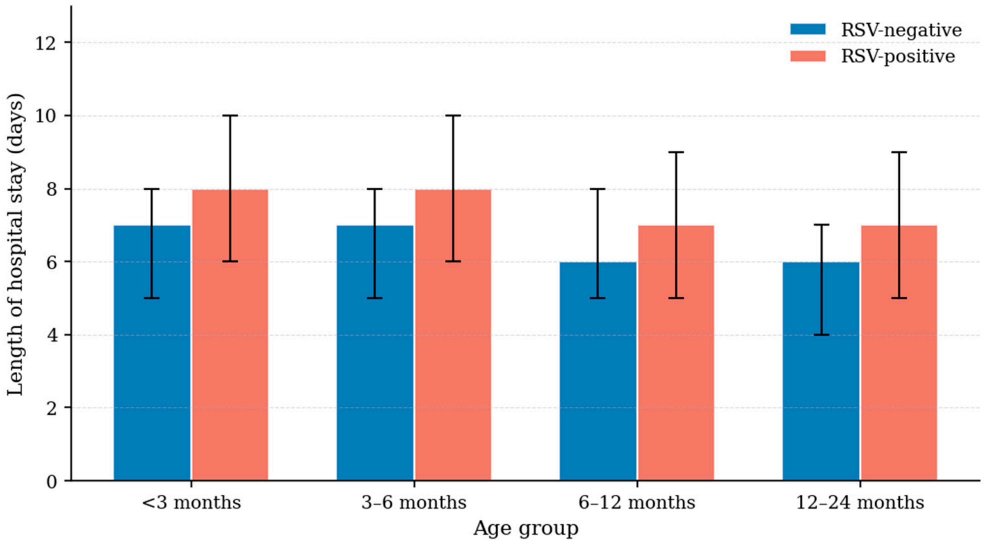

Figure S1. Length of hospital stay (LOS) by age group and RSV status among RSV-tested patients. Bars represent median LOS in days; error bars indicate interquartile range (IQR). RSV-positive patients consistently showed longer hospital stays across all age groups ($p < 0.001$, Mann–Whitney U test).

Table S2. Length of hospital stay according to treatment administered.

Treatment	LOS if not administered, median (IQR)	LOS if administered, median (IQR)
Oxygen therapy	6 (5–8)	8 (6–10)
Corticosteroids	6 (5–8)	7 (5–9)
Antibiotics	6 (5–8)	8 (6–10)
Nebulized therapy	6 (5–8)	7 (5–9)

Note: *LOS: length of hospital stay, expressed as median (interquartile range). All comparisons performed using the Mann–Whitney U test; p < 0.001 for all treatments.*

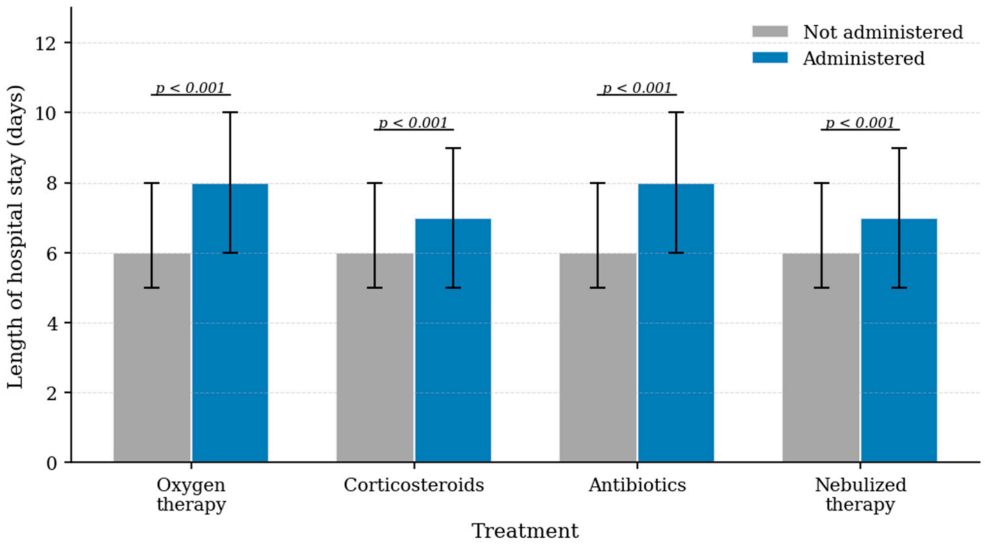

Figure S2. Length of hospital stay (LOS) according to treatment administered. Bars represent median LOS in days; error bars indicate interquartile range (IQR). Patients who received oxygen therapy or antibiotic therapy had a significantly longer hospital stay compared to those who did not (p < 0.001, Mann–Whitney U test).
